# Surgical Management of Concurrent Septic Arthritis and Forearm Necrotizing Fasciitis in an Immunocompromised Patient With a 20-Year History of Chronic Lymphocytic Leukemia: A Case Report

**DOI:** 10.7759/cureus.74739

**Published:** 2024-11-29

**Authors:** Burak Ozturk, Arın Celayir, Ali Osman G Cibikci, Cumhur Deniz Davulcu, Gokhan Kaynak

**Affiliations:** 1 Orthopaedics and Traumatology, Istanbul University Cerrahpasa - Cerrahpasa Faculty of Medicine, Istanbul, TUR

**Keywords:** cll, fasciotomy, gas gangrene, necrotizing fasciitis, septic arthritis

## Abstract

Shoulder septic arthritis is a severe infection of the shoulder joint, commonly caused by bacteria such as *Staphylococcus aureus*. It leads to inflammation, severe pain, swelling, and reduced mobility in the affected shoulder. The condition is typically diagnosed through clinical evaluation, blood tests, imaging studies, and joint aspiration. Prompt treatment, usually involving antibiotics and sometimes surgical intervention, is crucial to prevent joint destruction and other serious complications.

Necrotizing fasciitis of the upper extremity is a rapidly progressing, life-threatening bacterial infection that affects the fascia - the connective tissue surrounding muscles, nerves, and blood vessels. This condition is characterized by severe pain, swelling, erythema, and tissue necrosis. Commonly caused by bacteria such as *Streptococcus pyogenes*, it requires immediate medical attention. Diagnosis is often based on clinical evaluation, imaging, and laboratory tests.

We discuss the surgical treatment of a patient referred to our clinic with a preliminary diagnosis of septic arthritis and a history of chronic lymphocytic leukemia that had been untreated and not followed up. During the examination, we also considered the possibility of gas gangrene in the arm and forearm.

## Introduction

Septic arthritis of the shoulder joint is a severe and potentially debilitating condition characterized by the infection of the synovial fluid and tissues within the shoulder joint. This infection is typically caused by bacteria such as *Staphylococcus aureus* and can lead to rapid joint destruction and significant functional impairment if not promptly diagnosed and treated [1]. Risk factors include pre-existing joint conditions, immunosuppression, recent joint surgery, or trauma. Clinical presentation often includes acute onset of severe shoulder pain, swelling, warmth, and restricted range of motion. The treatment of shoulder joint septic arthritis involves a prompt and aggressive intervention to eradicate the infection and preserve joint function. Initially, empirical broad-spectrum intravenous antibiotics are administered, which are later tailored to culture results once the causative organism is identified [2]. In addition to antibiotic therapy, surgical intervention is often necessary. This may include arthroscopic or open surgical drainage and debridement to remove infected synovial fluid and necrotic tissue. In severe cases, repeated washouts or more extensive surgical procedures may be required [3].

Necrotizing fasciitis of the arm and forearm is a life-threatening bacterial infection characterized by the rapid destruction of fascia, fat, and muscle tissues. Commonly referred to as a "flesh-eating" infection, it often begins with minor trauma or an invasive procedure, allowing bacteria like *Streptococcus pyogenes* or *Clostridium perfringens* to invade. Symptoms include severe pain, swelling, erythema, and the presence of gas in tissues, potentially progressing to systemic toxicity and shock [4]. Prompt diagnosis and aggressive treatment are crucial, involving high-dose intravenous antibiotics and urgent surgical debridement to remove necrotic tissue. Multiple surgical interventions may be necessary to fully control the infection [5]. In some cases, necrotizing fasciitis can spread to adjacent joints, leading to septic arthritis, and septic arthritis can also progress to necrotizing fasciitis through the spread of infection across anatomical planes.

We discuss the surgical treatment of a patient referred to our clinic with a preliminary diagnosis of septic arthritis in the context of untreated and unmonitored chronic lymphocytic leukemia (CLL). During the examination, we also identified the potential for gas gangrene in the arm and forearm.

## Case presentation

**Figure 1:**
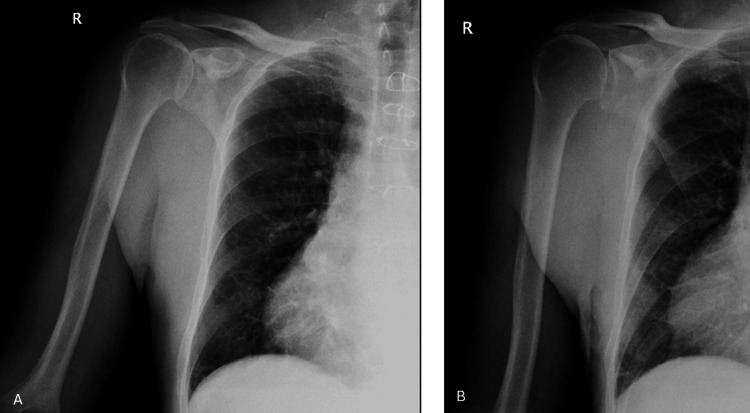
X-ray images of the patient’s right shoulder taken upon arrival. (A) Anteroposterior (AP) view of the shoulder; (B) True AP view of the shoulder.

A 76-year-old male patient presented with complaints of pain and restricted movement in his right shoulder. The patient had a known 20-year history of CLL for which he had not received any treatment. Upon arrival at the emergency department, X-rays of the shoulder were obtained (Figure 1).

**Figure 2:**
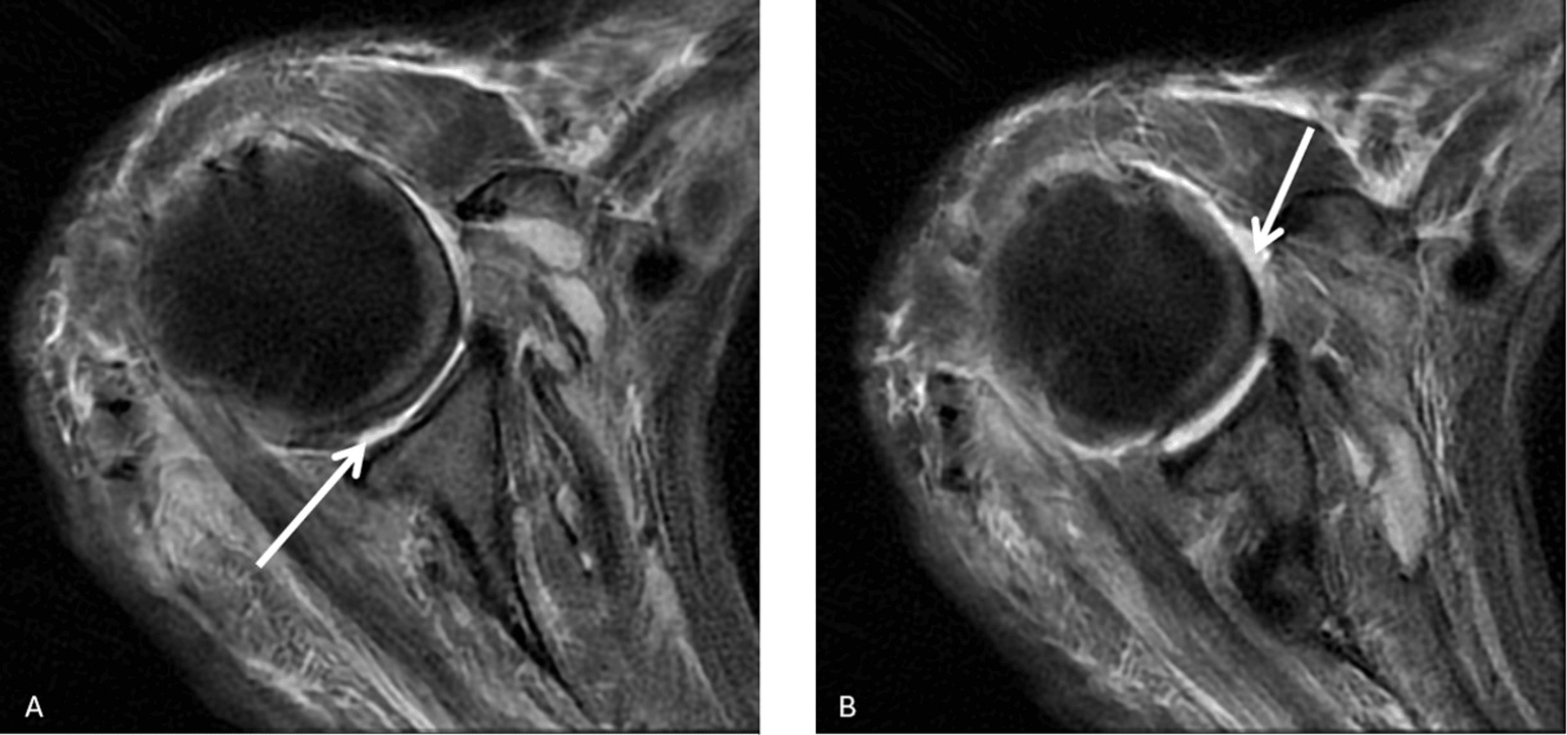
Axial imaging sections of the patient's right shoulder. (A, B) Arrows indicate the effusion surrounding the shoulder joint.

**Figure 3:**
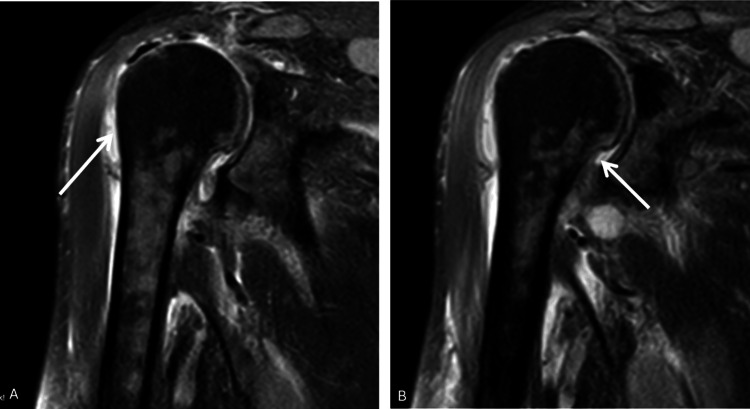
Sagittal imaging sections of the patient's right shoulder. (A, B) Arrows highlight the effusion surrounding the shoulder joint.

**Figure 4:**
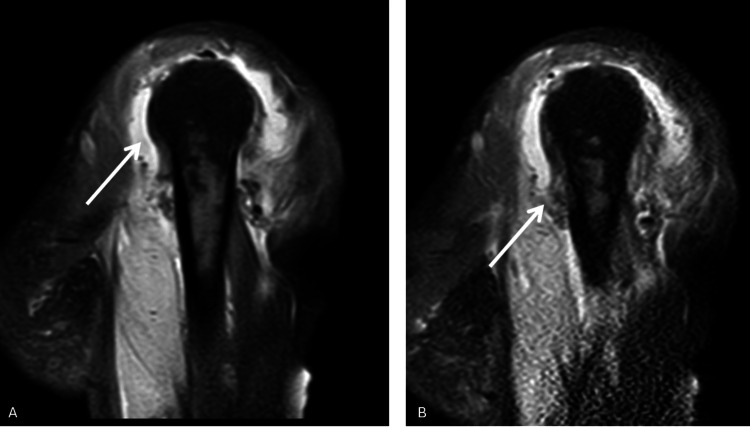
Coronal imaging sections of the patient's right shoulder. (A, B) Arrows highlight the effusion surrounding the shoulder joint.

Informed consent was obtained before any procedures were performed. During the examination, the patient exhibited significant movement restriction in the right shoulder. Palpable lymph nodes were noted in the left axillary region and bilaterally in the lateral neck areas. Additionally, the patient experienced crepitus and pain upon pressure in the medial aspect of the right arm and forearm. At presentation, acute phase reactants were markedly elevated: the white blood cell (WBC) count was 44,800, procalcitonin was 1.61 ng/mL, and C-reactive protein (CRP) was 314 mg/L. The patient's Laboratory Risk Indicator for Necrotising Fasciitis (LRINEC) score was calculated as 9, which, according to the scoring system, indicates a high risk. An urgent contrast-enhanced MRI was performed, which revealed widespread effusion within the shoulder joint and findings consistent with extensive anaerobic infection around the biceps tendon in the proximal arm (Figures 2-4).

**Figure 5:**
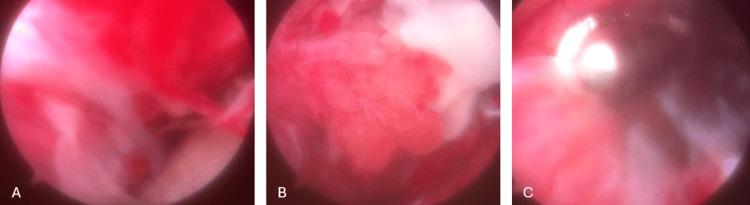
Arthroscopic images of the patient’s shoulder. Synovial fraying is evident in all three figures.

A puncture was performed on the right shoulder joint, and gram-negative bacilli growth was detected. The patient underwent arthroscopic washing, open debridement, and fasciotomy in collaboration with the plastic surgery team during the same session. Arthroscopic examination of the joint revealed widespread synovial fraying (Figure 5).

**Figure 6:**
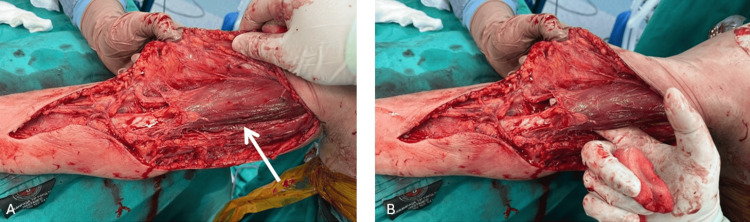
Intraoperative images captured during the fasciotomy procedure of the arm and forearm. The arrows indicate the biceps muscle.

No purulent discharge was observed within the shoulder joint. After debridement, the plastic surgery team performed a fasciotomy on the right arm and forearm. Necrotic tissues surrounding the biceps muscle were excised, but the biceps muscle itself was not removed. No additional debridement procedures were performed after the fasciotomy. Daily sterile surgical dressings were applied in the operating room (Figure 6).

**Figure 7:**
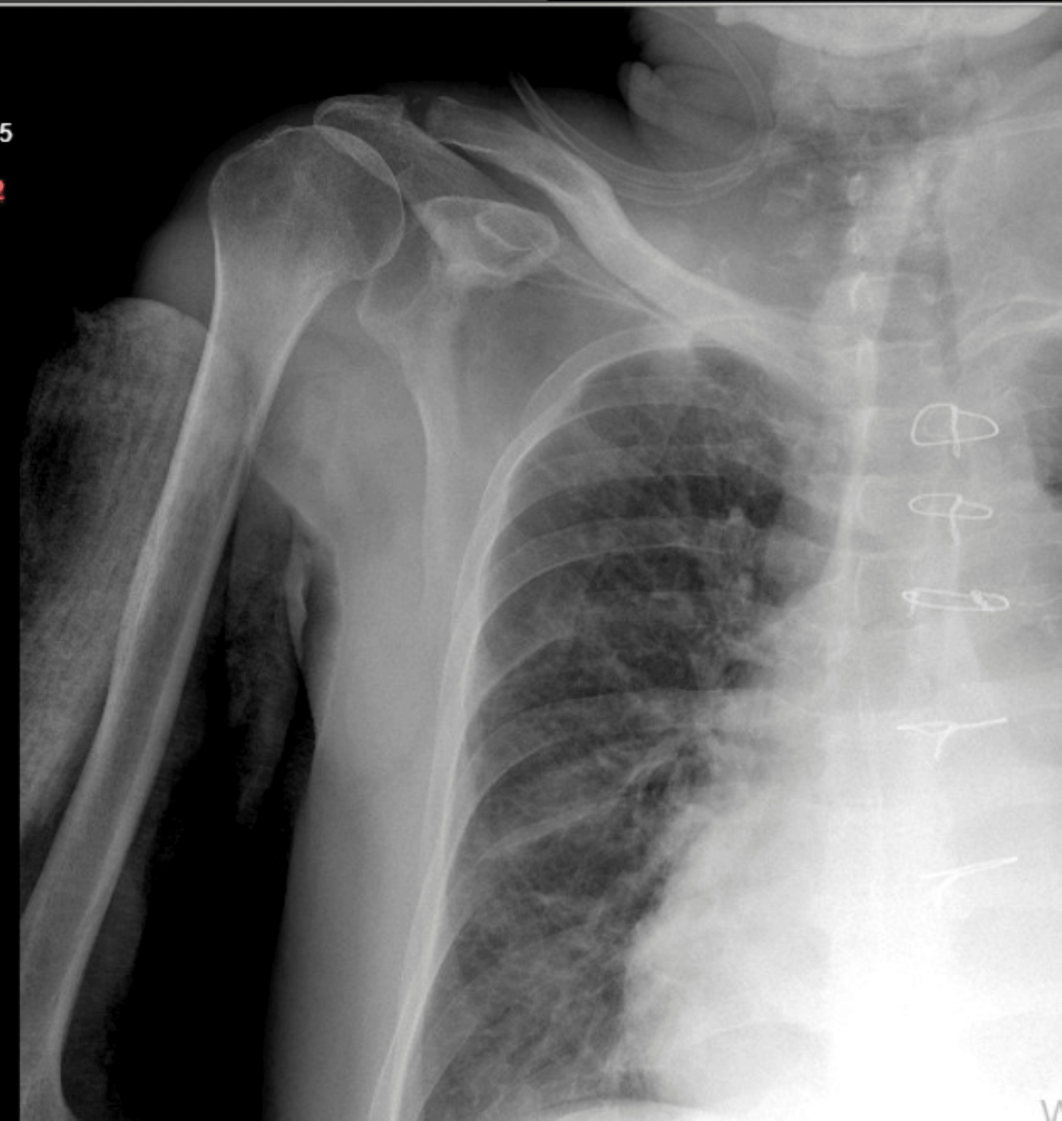
Postoperative X-ray images of the patient's right shoulder.

Postoperatively, the patient was transferred to the internal medicine intensive care unit for close monitoring (Figure 7).

Extended cultures revealed the growth of *Enterobacter*, prompting consultation with the infectious diseases team. The patient initially exhibited growth of gram-positive diplococci in cultures obtained via aspiration, but prolonged cultures and samples taken during surgery revealed the growth of gram-negative bacilli. Accordingly, the patient received intravenous antibiotic therapy. Initially, the patient was treated with vancomycin, clindamycin, and meropenem. However, following the growth of gram-negative bacilli in prolonged cultures, vancomycin was discontinued.

A summary of the procedures performed on the patient is presented in the form of a timetable (Table 1).

**Table 1:** Timeline summarizing the procedures performed on the patient.

Day/hour	Clinical event
Day 1 - Admission	Presentation with right shoulder pain, movement restriction, and palpable lymph nodes. Initial X-ray and physical examination findings raised suspicion for infection.
Day 1 - Admission 0-2 Hours	Laboratory results revealed elevated WBC count, procalcitonin, and CRP. MRI performed, showing widespread effusion and anaerobic infection around the biceps tendon.
Day 1 - Admission 6-10 Hours	Emergency surgical intervention and initiation of high-dose intravenous antibiotics.
Day 1 - Admission 12-24 Hours	Postoperative management included wet-to-dry dressings and infection monitoring to address necrotizing fasciitis.
Days 2-7	Clinical improvement observed, with reduction in inflammatory markers and pain.

## Discussion

Septic arthritis of the shoulder joint is a serious condition characterized by infection of the synovial fluid and tissues, typically caused by bacteria such as *S. aureus*. Without prompt treatment, it can rapidly destroy the joint and impair function. Risk factors include prior joint conditions, immunosuppression, recent surgery, or trauma. Symptoms often include sudden onset of shoulder pain, swelling, warmth, and restricted movement. Treatment requires urgent action to clear the infection and preserve joint function. Initial management includes broad-spectrum intravenous antibiotics, adjusted based on culture results [6]. Surgery, such as arthroscopic or open drainage with tissue debridement, is often necessary to manage severe cases and prevent further damage.

Our patient had a 20-year history of CLL. Although the patient had no significant trauma, the presence of crepitus and pain in the arm and forearm during examination prompted a change in our treatment approach, leading to surgery in collaboration with the plastic surgery team. Cultures did not show growth of the most common pathogen, including *S. aureus*. However, extended cultures revealed the growth of *Enterobacter*. Consequently, the patient underwent a six-week inpatient intravenous antibiotic regimen.

Simultaneous occurrence of shoulder septic arthritis and arm necrotizing fasciitis is an uncommon but serious clinical scenario. Shoulder septic arthritis involves bacterial infection within the shoulder joint, which can lead to rapid joint destruction if left untreated. In contrast, arm necrotizing fasciitis is a severe soft tissue infection characterized by rapidly spreading necrosis of the fascial planes. Both conditions require urgent surgical intervention and aggressive antibiotic therapy to halt the infection, remove necrotic tissue, and preserve limb function. Management requires close collaboration between orthopedic and plastic surgery teams to provide comprehensive treatment and optimize outcomes. Although definitive evidence regarding the initial source of infection was lacking in our patient, presenting with shoulder pain and subsequent sensitivity in the arm and forearm raised suspicion of complicated necrotizing fasciitis secondary to shoulder infection. Given the aggressive clinical progression in both areas, the patient underwent immediate surgery. Postoperative assessment revealed regression in acute phase reactants.

Untreated or poorly controlled CLL poses significant challenges in patient management [7]. This condition, characterized by the proliferation of abnormal lymphocytes, compromises the immune system and increases susceptibility to infections [8]. The rapid progression of our patient’s clinical condition, necessitating intensive care, was primarily attributed to immunosuppression from untreated CLL. The patient’s overall condition deteriorated significantly within one day. Postoperatively, the patient was closely monitored in the intensive care unit for an extended period.

## Conclusions

The surgical treatment of shoulder septic arthritis and upper extremity necrotizing fasciitis requires a coordinated approach to prevent complications and improve outcomes. It is rare for both conditions to occur together, but if they do, prompt surgical intervention and appropriate antibiotic therapy are crucial.
